# Impact of Prebiotics and Synbiotics Administered *in ovo* on the Immune Response against Experimental Antigens in Chicken Broilers

**DOI:** 10.3390/ani10040643

**Published:** 2020-04-08

**Authors:** Tadeusz Stefaniak, Jan P. Madej, Stanisław Graczyk, Maria Siwek, Ewa Łukaszewicz, Artur Kowalczyk, Marcin Sieńczyk, Giuseppe Maiorano, Marek Bednarczyk

**Affiliations:** 1 Wroclaw University of Environmental and Life Sciences, Department of Immunology, Pathophysiology and Veterinary Preventive Medicine, 50-375 Wroclaw, Poland; tadeusz.stefaniak@upwr.edu.pl (T.S.); stanislaw.graczyk@upwr.edu.pl (S.G.); 2 Wroclaw University of Environmental and Life Sciences, Division of Histology and Embryology, 50-375 Wroclaw, Poland; 3 UTP University of Science and Technology, Department of Animal Biotechnology and Genetics, 85-796 Bydgoszcz, Poland; siwek@utp.edu.pl (M.S.); marbed13@op.pl (M.B.); 4 Wrocław University of Environmental and Life Sciences, Institute of Animal Breeding, 51-630 Wrocław, Poland; ewa.lukaszewicz@upwr.edu.pl (E.Ł.); artur.kowalczyk@upwr.edu.pl (A.K.); 5 Wroclaw University of Technology, Division of Medicinal Chemistry & Microbiology, 50-372 Wroclaw, Poland; marcin.sienczyk@pwr.edu.pl; 6 University of Molise, Department of Agricultural, Environmental and Food Sciences, 86100 Campobasso, Italy; maior@unimol.it

**Keywords:** *in ovo*, delayed-type hypersensitivity, SRBC, dextran, immunoglobulin

## Abstract

**Simple Summary:**

The immune system of chickens matures in the course of embryonic development. Early *in ovo* supplementation with bioactive substances leads to the long-term maintenance of a high level of intestine bifidobacteria, reduces the number of detrimental microorganisms in the gut, modulates the central and peripheral lymphatic organ development in broilers, and stimulates gut-associated lymphoid tissues (GALT) development after hatching. In this investigation, we studied whether the early *in ovo* application (at the 12th day of embryo incubation) of selected bioactives (prebiotics and synbiotics) influences the humoral immune response against experimental antigens, and the delayed-type hypersensitivity (DTH) skin reaction to experimental mitogen. This study demonstrated that the *in ovo* application of bioactives did not significantly influence the humoral immune response against T-dependent and T-independent model antigens. Prebiotics in chickens immunized with T-dependent antigen (SRBC) protected them from a retarded rise of the IgG concentration. Bioactives reduced the mortality of birds, markedly with inulin (−6.4%), and the DTH reaction to phytohemagglutinin on the 7^th^ and 21^st^ day after hatching.

**Abstract:**

The effect of the *in ovo* application of selected prebiotics and synbiotics on the humoral immune response against T-dependent (SRBC) and T-independent (dextran) antigens and delayed-type hypersensitivity (DTH) to phytohemagglutinin was studied. On the 12th day of incubation, 800 eggs (Ross 308) were divided into five groups and injected into the egg air chamber with prebiotic inulin (Pre1), Bi^2^tos (Pre2), a synbiotic composed of inulin and *Lactococcus lactis* subsp. *lactis* IBB SL1 (Syn1), a synbiotic composed of Bi^2^tos and *L. lactis* subsp. *cremoris* IBB SC1 (Syn2), and physiological saline (control group; C). The chickens were immunized twice at the 7th and 21st day of life with SRBC and dextran. A DTH test was performed on the 7th, 21st, and 35th day. The application of prebiotics and synbiotics had no significant effect on the humoral immune response. SRBC-immunized *in ovo* Pre1- and Pre2-treated chickens showed significantly higher serum IgG levels than the control. A significant effect on the DTH reaction was detected on the 7th (Pre1 < C) and 21st (Pre2 > Syn2) day. However; Bi^2^tos may transiently stimulate the cellular immune response on the 21st day. It may be concluded that the application of inulin in an egg air chamber on the 12th day of incubation may stimulate the secondary immune response. The inulin-treated group exhibited a lower mortality rate than the control group.

## 1. Introduction

Many beneficial effects of the dietary application of nondigestible carbohydrates (prebiotics) in poultry have been well-documented [1,2,3,4,5,6]. Inulin fermentation products in the gut have anti-inflammatory effects [7]. Antigenic fragments of probiotics given orally can modulate the innate and adaptive immune response [8] by stimulating the intestinal immune system [9,10] and can also affect immune responses in other lymphatic organs [11]. Probiotics given orally to one-day-old chicks stimulate the cellular immune response to phytohemagglutinin (PHA) and dinitrochlorobenzene (DNCB) [12].

*In ovo* technology enables the administration of a given substance in a solution directly into the incubating eggs [13,14,15]. Day 12 of incubation has been estimated as the optimal time for prebiotic injection into the air chamber of the incubating egg [15,16]. Early *in ovo* supplementation with bioactive substances (on the 12th day of chicken embryo development) leads to the long-term maintenance of a high level of intestine bifidobacteria, reduces the number of detrimental microorganisms in the gut [13,17,18], modulates the central and peripheral lymphatic organ development in broilers [19], and stimulates gut-associated lymphoid tissues (GALT) development after hatching [20,21]. Galactooligosaccharides delivered *in ovo* mitigated heat-stress-induced chronic systemic oxidative stress and decreased the Th2 response in slow-growing chickens [22]. Selected synbiotics given *in ovo* can modulate the development of immune organs, e.g., the development of bursa of Fabricius and the spleen, and lymphocyte proliferation in the thymus [23,24], or downregulate the immune-related gene expression in the cecal tonsils and spleen in chickens [25]. However, the mechanisms underlying the interaction between prebiotics and synbiotics and the host immune system are not known.

Therefore, the aim of this study was to evaluate whether the early *in ovo* application (at the 12th day of embryo incubation) of selected prebiotics and synbiotics influences the humoral immune response against experimental antigens and the delayed-type hypersensitivity (DTH) skin reaction to experimental mitogen.

## 2. Materials and Methods

### 2.1. Materials

The source material was described in Stefaniak et al. [26]. The scheme of the experiment, including the division of embryos and chickens into experimental groups, is shown in Table 1 and Table 2.

### 2.2. In ovo Treatment

The experiments were conducted with the consent of the Local Ethical Committee for Animal Experiments (24/2011, Bydgoszcz, Poland). The eggs were incubated at a commercial hatchery (Drobex, Solec Kujawski, Poland) in a Petersime incubator (Zulte, Belgium), using standard conditions (37.8 °C and a relative humidity of 61 ± 63%). The eggs were candled at the 12th day of incubation and those with developing embryos were used in the experiments. On the 12th day of incubation, the eggs were injected into the air chamber with 0.2 mL of solution/suspension of bioactive substances, as described in Stefaniak et al. [26]. Bioactive compounds (prebiotics or synbiotics) delivered *in ovo* were as follows: 1.76 mg/egg inulin prebiotic (Pre1) (Sigma-Aldrich GmbH, Schnelldorf, Germany); 0.528 mg/egg Galactooligosaccharides (GOS) prebiotic (trade name: Bi^2^tos) (Pre2) (Clasado Biosciences Ltd., Jersey, UK); synbiotic composed of 1.76 mg/egg inulin and 1000 CFU/egg *Lactococcus lactis* subsp. *lactis* IBB SL1 (Syn1); or a synbiotic composed of 0.528 mg/egg GOS and 1000 CFU/egg *Lactococcus lactis* subsp. *cremoris* IBB SC1 (Syn2). These synbiotics were selected from several combinations of pre- and probiotics by in vitro tests, followed by validation with an animal model [27,28]. The control group (C) was injected with physiological saline.

### 2.3. Rearing Conditions

The rearing conditions were described in Stefaniak et al. [26]. The feed was free from pre- and probiotics and antibiotics.

### 2.4. Collecting the Yolk Sac Content

On the 12th day of incubation (E12), from the pool of incubated eggs, seven eggs were randomly selected and opened with scissors, the embryos were killed by decapitation, and the yolk sac content was taken. Subsequently, on the 18th day of incubation (E18), seven eggs were randomly selected from each experimental group, and the procedure was the same as on E12. On the day of hatching (D1) and on the 4th (D4) day of life, seven chickens from each group at both time points were randomly selected, weighed, and killed by decapitation. The yolk sac content collected both from embryos and chicks was diluted to a ratio of 1:4 with phosphate-buffered saline (PBS, pH 7.3) and stored at −22 °C until further use.

### 2.5. Immunization

On day 7 of age (D7), randomly selected chickens from each of the five groups (C, Pre1, Pre2, Syn1, and Syn2) were divided into three subgroups: (1) SRBC—birds injected intramuscularly with 200 µL of a 5% suspension of sheep red blood cells (SRBC) (Pro Animali, Wroclaw, Poland); (2) DEX—birds immunized subcutaneously with 200 µL of solution containing 1 mg of dextran (molecular weight 5–40 MDa; Koch Light Laboratories, Ltd., Haverhill, Suffolk, UK) in PBS; and (3) N—nonimmunized, destined for DTH testing and IgG estimation. The immunization procedure was repeated within groups on day 21 of age (D21) (Table 2).

### 2.6. Blood Sampling and Serum Preparation

From seven chickens of each group on D1, D4, and D7, blood from the cervical vein was sampled immediately after killing. On D21 and D35, the blood was taken by the same procedure from seven chickens of 15 groups. Each of the five *in ovo*-treated groups (C, Pre1, Pre2, Syn1, and Syn2) was divided into three subgroups: N—no additional treatment, DEX—immunized with dextran, and SRBC—immunized with SRBC (Table 1 and Table 2). The serum was separated by centrifugation at 2000× *g* for 8 min and stored at −20 °C until use.

### 2.7. Evaluation of the IgG Concentration in Chicken’s Serum and Yolk Sac Content

The chicken IgG concentration in the yolk sac (IgY) and blood serum was estimated using radial immunodiffusion, according to Gąsowska and Stefaniak [29]. Blood serum was undiluted, whereas the yolk sac content was diluted with PBS (pH 7.3) at a ratio of 1:4. A standard curve (0.1, 0.25, 0.5, 1, 2, 5, and 10 g of chicken IgG/L) was produced based on chicken IgG (Sigma) diluted in sheep serum albumin (70 g/L).

### 2.8. Determination of the SRBC antibody (against T-dependent antigens)

The SRBC antibody was determined by microagglutination (modified by Hebishima et al. [30]). Serum samples taken on D7, D21, and D35 were inactivated by incubation at 56 °C for 30 min. The serum samples were serially diluted with PBS in twofold steps (1:1–1:128) in U-bottomed microplates (96 well, MedLab), with 100 µL/well. In the next step, 25 µL of 2% SRBC suspension was added to each well. The plates were incubated for 18 h at 4 °C and at room temperature concurrently. The results were evaluated as follows: 0, no agglutination; ±, doubtful; +, weak agglutination; ++, marked net of agglutinated erythrocytes; +++, strong agglutination with curled-up borders. The numerical reverse result of the highest positive dilution was calculated by the addition of the reverse titer 0.5, 1, 2, or 3, respectively, to the intensity ±, +, ++, or +++ of the highest positive dilution.

### 2.9. Determination of IgG and IgM Dextran Antibodies Using ELISA

To determine the immune response of chickens against T-independent antigens in ELISA, dextran was chosen [31]. Ninety-six-well microplates (F-bottomed, Nunc) were coated with dextran (molecular weight of 5–40 MDa; Koch Light Laboratories, Ltd., UK) diluted in 0.15 M carbonate buffer at a pH of 9.6 (50 µL/well dextran dilution (1 mg/mL)) and incubated for 3 h at 37 °C and overnight at 4 °C. Microplates were washed three times with PBS at pH 7.3 containing 0.05% Tween 20 (microplate washer Biotek EL × 50). Subsequently, serum diluted at a ratio of 1:100 was added (50 μL/well, in two repetitions for each antibody class) and microplates were incubated for 2 h at room temperature under stirring (ELPAN Laboratory Stirrer). The plates were then washed as described above and 50 μL/well of the rabbit anti-chicken IgG (Sigma, 1:20,000) or goat anti-chicken IgM (Sigma, 1:20,000) horseradish peroxidase (HRP) conjugates was added and incubated for 2 h at room temperature under stirring. The plates were washed again as described above and substrate solution (100 μL, TMB, Sigma) was added and incubated at room temperature in the dark for 20 min. The reaction was stopped by adding 25 μL 2M H_2_SO_4_/well. The absorbance was read at 450 nm (Microplate reader BioTek µQuant).

### 2.10. Evaluation of Mitogen-Induced Cutaneous Delayed-Type Hypersensitivity (DTH)

The cutaneous hypersensitivity to T-cell mitogen (phytohemagglutinin, PHA) was evaluated according to Kean and Lamont [32], as modified by Graczyk et al. [33]. On D7, D21, and D35, eight chickens from each of the five groups were randomly selected and injected with 100 µg PHA (Sigma) dissolved in 100 µL PBS at pH 7.2. The injections were performed intradermally into the skin between the third and fourth digits of the right foot on D7. An identical dose of PHA was administered into the wing web of the right wing to 21-day-old and 35-day-old chickens. The PBS administered to the other wing was assumed as the control. The thickness of the skin fold, found exactly at the place of injection of the PBS or PHA, was measured before injection and 24 h after. Measurements were repeated three times, using a constant-loading dial micrometer (Mitutoyo, Japan). The response level was determined by calculating the wing index (WI) as the difference in the fold thickness before and 24 h after the intradermal injection.

### 2.11. Statistical Analysis

Quantitative data were subjected to statistical analysis using Statistica 10.0 software (StatSoft Polska Sp. z o.o., Kraków, Poland). The significance of differences between the results obtained was appraised using a one-way ANOVA test for data showing a normal distribution and a Kruskal–Wallis ANOVA test for data that were not normally distributed. Where one-way ANOVA was statistically significant, subgroup analysis was performed using Tukey’s honest significant difference test. The normal distribution revealed the serum IgG concentration, serum reverse agglutination titer against SRBC, serum anti-dextran IgM/IgG antibody levels, and DTH. The significance of differences in mortality was appraised using a two-sample z test. A value of *p <* 0.05 was considered significant.

## 3. Results

### 3.1. Yolk Sac IgG (Y) Concentration

**Figure 1:**
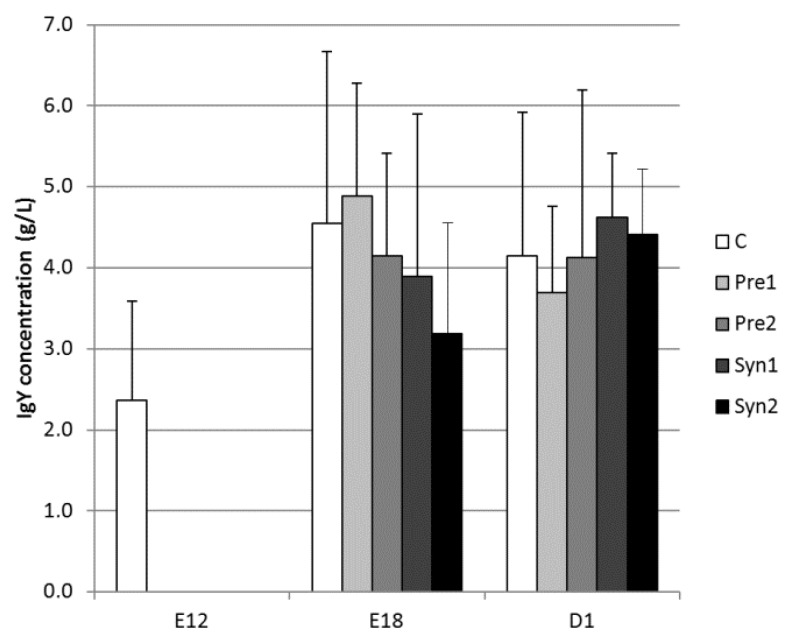
Yolk sac IgG (Y) concentration (mean ± SD) in chickens between the 12th day of embryo development (E12) and 1st day after hatching (D1): C – control, Pre1 – prebiotic 1, Pre2 – prebiotic 2, Syn1 – synbiotic 1, and Syn2 – synbiotic 2.

The yolk sac IgG (Y) concentration in the C group increased slightly between E12 and E18 (2.37 versus 4.54 g/L, respectively; *p* = 0.076), and an intermediate value was observed in the D1 group (*p* > 0.05). There were no significant differences in IgG values between E18 and D1 for all experimental groups, except for the Syn2 (4.41 versus 3.19 g/L for D1 and E18, respectively; *p* = 0.065). There were no statistically significant differences in IgG values between the experimental groups within the individual estimated periods (E18 and D1) (Figure 1).

### 3.2. Serum IgG Concentration

**Figure 2:**
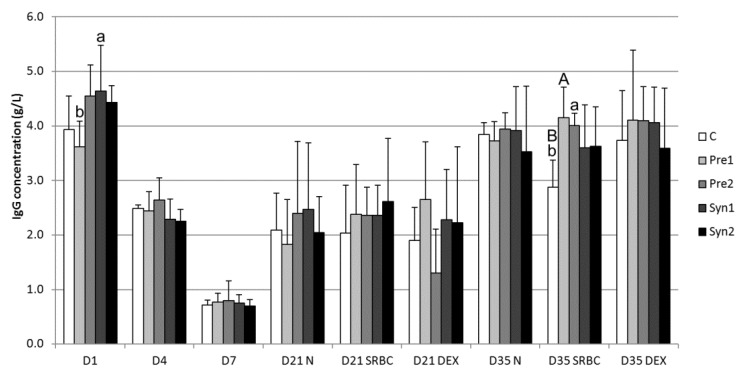
Serum IgG concentration (mean ± SD) in chickens between the 1st day (D1) after hatching and 35th (D35) day of life: C – control, Pre1 – prebiotic 1, Pre2 – prebiotic 2, Syn1 – synbiotic 1, Syn2 – synbiotic 2, N – untreated groups, SRBC – SRBC-treated groups, and DEX – dextran-treated groups; significant difference A,B: *p* < 0.01; a,b: *p* < 0.05. The differences between D1 and D7 in the relevant groups were significant (*p* < 0.001), as well as between D7 and D35N (*p* < 0.01).

The results of the total serum IgG (g/L) concentration are presented in Figure 2. In all the experimental groups, the concentration of total serum IgG decreased (*p* < 0.001) from D1 to D4 and from D4 to D7, and then increased between D7 and D21 (*p* < 0.01) and from D21 to D35 (*p* < 0.05 and *p* < 0.01). In fact, the serum IgG concentration at the hatching time was approximately 4 mg/mL in all the groups, but decreased stepwise on D4 (about 2.5 mg/mL) and D7 (below 1 mg/mL). In addition, at the hatching time (D1), the serum IgG concentration was higher in the Syn1 group when compared to the Pre1 group (4.63 versus 3.62 g/L; *p* < 0.05), while no significant differences were found in IgG values among the experimental groups at the time points of D4 and D7. Concentrations of IgG among the non-treated (without SRBC or DEX stimulation) groups at each time point considered (D21N and D35N) did not differ significantly (*p* > 0.05). Furthermore, immunization with SRBC did not influence the IgG concentration in both prebiotic- and synbiotic-treated groups on D21. On the contrary, on D35, the IgG level increased in prebiotic- and synbiotic-immunized groups. However, this increase was only evident in prebiotic groups compared to the control (2.97 versus 4.15 mg/L for control and Pre1 groups, respectively; *p* < 0.01. 2.97 versus 4.0 mg/L for control and Pre2 groups, respectively; *p* < 0.05). The immunization with dextran did not affect (*p* > 0.05) the IgG concentrations in all groups and at all time points.

### 3.3. Anti-SRBC and Anti-Dextran Antibodies

**Figure 3:**
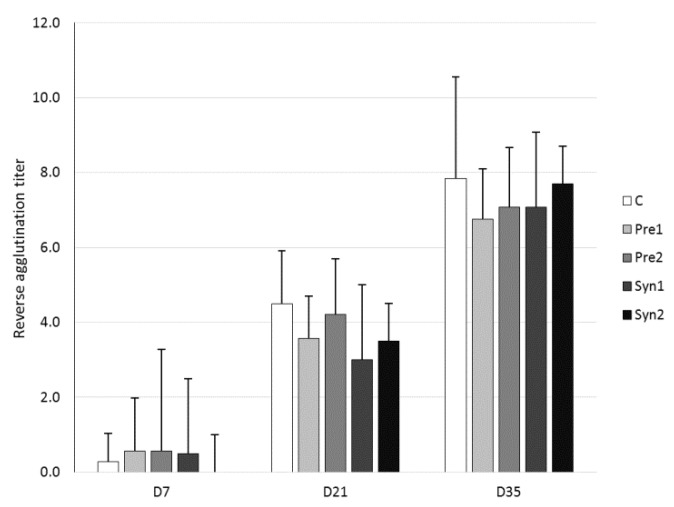
Serum reverse agglutination titer (mean ± SD) against SRBC: C – control, Pre1 – prebiotic 1, Pre2 – prebiotic 2, Syn1 – synbiotic 1, and Syn2 – synbiotic 2.

**Figure 4:**
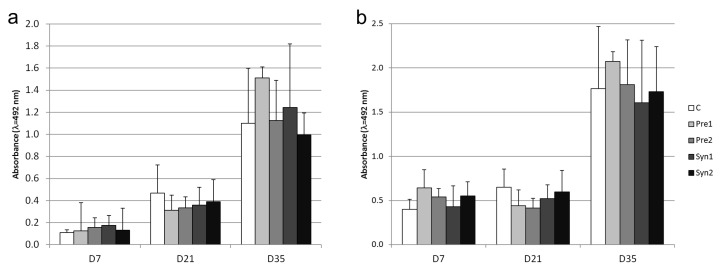
Serum anti-dextran IgM (**a**) and anti-dextran IgG (**b**) antibody level (mean ± SD) in chickens immunized with dextran: C – control, Pre1 – prebiotic 1, Pre2 – prebiotic 2, Syn1 – synbiotic 1, and Syn2 – synbiotic 2; D7 – 7th day, D21 – 21st day, and D35 – 35th day.

The results of the serum reverse agglutination titer against SRBC are reported in Figure 3. In all the experimental groups, the concentration of serum reverse agglutination titers increased from D7 to D21 and then from D21 to D35 (*p* < 0.05 and *p* < 0.001). Conversely, no significant differences (*p* > 0.5) were found in the serum reverse agglutination titer values among the experimental groups at each time point (D7, D21, and D35). The intensity of the serum IgM antibody reaction in DEX-treated groups was found to be higher on D35 than that on D7 (*p* < 0.01) and D21 (*p* < 0.05). In contrast, the serum IgM antibody values among experimental groups at each time point (D7, D21, and D35) did not significantly differ (*p* > 0.05) (Figure 4a). A similar trend was observed for the serum IgG anti-dextran antibodies concentration, for which the values were higher for each treatment on D35 with respect to D7 and D21 (*p* < 0.01), whereas the intensity of the IgG reaction did not differ (*p* > 0.05) among groups at the respective time points (Figure 4b).

### 3.4. Delayed-Type Hypersensitivity (DTH)

**Figure 5:**
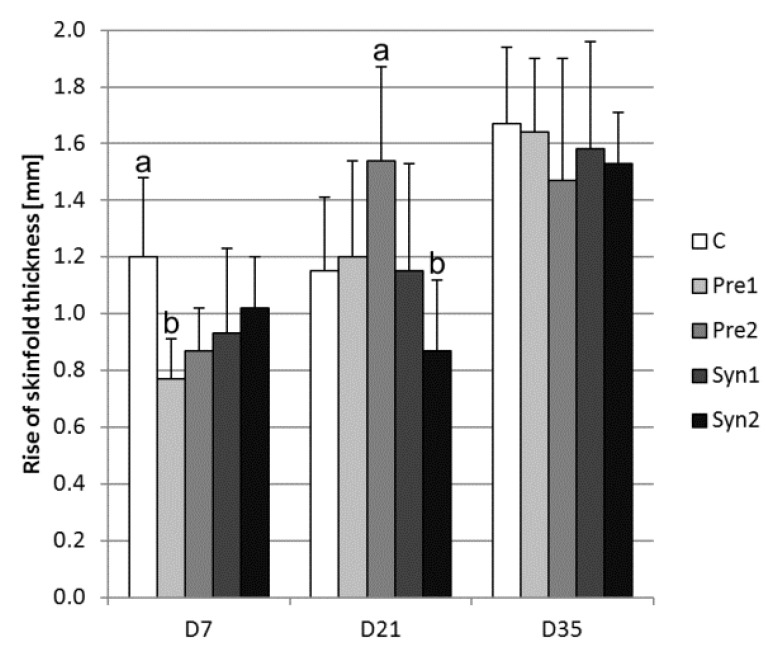
Delayed-type hypersensitivity (DTH) measured as an increase in skin fold thickness 24 h after an intradermal injection of phytohemagglutinin (mean ± SD): C – control, Pre1 – prebiotic 1, Pre2 – prebiotic 2, Syn1 – synbiotic 1, and Syn2 – synbiotic 2. Significant difference a,b; * *p* < 0.05 within time groups.

The DTH reaction was more marked from D7 to D35 (*p* < 0.05 and *p* < 0.01); however, significant increases were also observed in Pre2 from D7 to D21 (*p* < 0.05) and in the control and Syn2 from D21 to D35 (*p* < 0.05 and *p* < 0.01). Within the time groups, at time D7, the Pre1 value was lower compared to the control (0.77 versus 1.20 mm; *p* < 0.05), and at time D21, Pre2 was higher than Syn2 (1.54 versus 0.87 mm; *p* < 0.05). No significant differences were found for DTH among experimental groups at time D35 (Figure 5).

### 3.5. Mortality Rate

The mortality rate was lower in inulin (Pre1) compared to the other groups; however, significant differences (*p* < 0.05) were only found between control and Pre1 groups (Table 3).

## 4. Discussion

The immune system of chickens matures during embryonic development. The first population of T cells leaves the thymus on the 6th day of incubation [34]. The majority of lymphoid cells present in the thymus on the 12th and 13th days possess the features typical of differentiated lymphocytes [35]. The second and third waves of T-cell migration from the thymus occur on the 12th day of incubation and at hatching [34]. The emigration of B lymphocytes from the bursa of Fabricius takes place on about the 18th day of incubation [34]. Therefore, in the light of the findings from the above studies, we studied whether the application of prebiotics or synbiotics, injected into the air chamber on the 12th day of incubation, was able to influence the further stages of immune system development. The acquired immune system was evaluated considering the yolk sac IgG (Y) and serum IgG concentrations, the DTH reaction upon an intradermal PHA injection, the humoral immune response, and the generation of antibodies against T-dependent (SRBC) and T-independent (dextran) antigens.

In the chicken spleen, IgM-secreting cells were first apparent by 3 days of age, while IgG- and IgA-secreting cells were not seen until 6 days after hatching [36]. Kaspers et al. [37] detected that during the late phase of embryonic development, IgA can be transported from albumen into the yolk sac, thus increasing the yolk protein concentration. Moreover, it might be speculated that changes in the dry matter content and volume of the yolk sac on the last days of incubation may influence the protein concentration, including the IgY concentration. At hatching time, the yolk sac IgG (Y) concentration of the chicks (Ross 308) measured in this study (ranging from 3.69 to 4.62 mg/mL, Figure 1) was three times lower than that found in one-day-old Hubbard Flex chicks [38]. The transfer of yolk immunoglobulins into the embryos’ blood can already be detected on the 11th day of incubation, but its efficiency remains low and increases rapidly from the 18th day of incubation [39]. Kramer and Cho [40] found that this transport process begins at a slow rate on around the 7th day of incubation and increases stepwise during the last three days before hatching. In fact, Kowalczyk et al. [41] found that on the 14th day of incubation, the transport of IgG from the yolk sac to chickens’ blood reached about 30 µg/day, and its capacity increased to 600 μg/day on the 18th day of incubation. However, from the more than 100 mg of IgG present in the yolk sac, less than 10% is transported into the chickens’ blood. The transfer of IgG (Y) from the yolk sac to chicken blood is dependent on FcRY (receptor specific for the chicken IgG (Y) Fc fragment), expressed in the yolk sac membrane [42]. The successive decrease (Figure 2) of serum IgG between the 1st (more than 4 mg/mL) and 7th (less than 1 mg/mL) day after hatching indicates the utilization of maternal IgG obtained through the yolk sac and no signs of marked IgG production by the chicken until D7. The decrease in yolk sac-derived serum IgG was probably due to the utilization of antibodies against environmental antigens, transport into mucosal membranes, and the biodegradation of proteins. Moreover, it is probable that the rapid increase in body weight (and therefore the increase of the plasma volume) in the examined broiler chickens might be an additional factor that decreased the serum IgG concentration. The rise of serum IgG on the 21st and 35th day of life could be associated with the immune response to environmental antigens. Kowalczyk et al. [41] found that the mean serum IgG concentration at the time of hatching in Leghorn chickens was slightly above 1 mg/mL; at the same time, Chrząstek and Wieliczko [38] detected a value of about 3 mg/mL in Hubbard Flex broiler chickens. This is a lower concentration than that found in the Ross 308 chickens examined in this study. Several authors have indicated different time points as the start of IgG and IgM synthesis in chicken. Khattab and Craig [43] demonstrated self IgG synthesis on the 11th day after hatching. Kaspers et al. [44] determined the start of IgM synthesis between the 2nd and 4th day after hatching and the start of IgG synthesis on the 2nd–7th day of life. The *in ovo* application of prebiotics probably protected the chickens immunized with T-dependent antigen (SRBC) from the slower rise of the IgG concentration (Pre1 and Pre2 D21SRBC versus Pre1 and Pre2 D35SRBC, respectively) that was observed in the control group (C D21SRBC versus C D35SRBC) (Figure 2).

Brisbin et al. [10] used an SRBC antigen as a measure of the antibody-mediated immune response in birds treated orally with *Lactobacillus acidophilus, Lactobacillus reuteri*, and/or *Lactobacillus salivarius*. Their findings indicated that the systemic humoral immune response can be modulated by oral treatment with lactobacilli, but it should be kept in mind that these bacteria may vary in their ability to modulate the immune response. A statistically significant impact on the SRBC antibody level was detected in birds supplemented with *L. salivarius.* The other two bacteria strains did not significantly affect the systemic immune response. The lactobacilli bacteria used in the experiment of Brisbin et al. [10] are members of the chicken intestinal microbiota. In the present study, the bacterial strains were given only once, *in ovo*, in the air chamber at E12, and were not identified as a natural component of the chicken microbiome [23,45]. The presence of a low agglutination titer on D7 (in individual chickens from each group before immunization), as observed in our study (Figure 3), indicates the occurrence of a natural antibody that cross reacts with SRBC [46]. Commensal bacteria in the intestine interact with cells of the gut-associated lymphoid tissues (GALT). Among the cells of the GALT, B-1 cells are considered significant since these cells are involved in the production of natural antibodies [9]. Haghighi et al. [9] showed that manipulation of the intestinal microbiota by the administration of probiotics can affect natural antibody production in chickens. Natural antibodies are considered to be a crucial immune barrier in the initial steps of the immune response before the antibodies against foreign antigens are generated [47]. Based on the literature and the results obtained, it might be suggested that the *in ovo* injection of synbiotics can affect natural antibody synthesis in chickens.

No statistically significant differences were observed regarding the rise of the IgG and IgM anti-dextran antibody between control and experimental groups. The rise of the antibody seemed to diminish after first immunization in prebiotic- and synbiotic-treated chickens on D21 (Figure 4). However, after second immunization (D35), Pre1 chickens showed moderately higher IgM and IgG anti-dextran antibody levels compared to the other groups. It may be suggested that the application of Pre1 in the egg air chamber on the 12^th^ day of incubation may stimulate the secondary immune response.

In the present study, the *in ovo* application of selected pre- and synbiotics affected the DTH skin reaction on D7 (Pre1 < C) and D21 (Pre2 > Syn2), while no significant differences were found for DTH on D35. Karimi Torshizi et al. [12] indicated that chickens fed with prebiotics in drinking water for 40 days showed an enhanced cellular response to a phytohemagglutinin injection. In our study, it was found that DTH after an intradermal PHA injection increased according to the age between D7 and D35. The *in ovo* application of both prebiotics and synbiotics inhibited the DTH reaction on D7 (Figure 5), but this decrease was more evident between the control group and Pre1 group. It should be mentioned that in our study, the age and method of application of prebiotics and synbiotics differed from the parameters described in other studies.

The mortality rate in treatment groups ranged from 2.1% to 5.8%, while in the control group, it was 8.5%—a value significantly higher than that of the inulin-treated group (Pre1). This might be connected to the positive effect of this prebiotic on the immune response and health condition (reviewed in [48]). In fact, inulin supplementation in monogastric animals may have indirect and direct effects. The indirect impact refers to the stimulation of development of healthy intestinal microbial strains, which in turn inhibit the proliferation of pathogenic bacteria that may cause infections and produce toxins harmful to organisms [49,50]. The direct effect influences the activity of phagocytic cells [26], as well as nonspecific mechanisms of humoral immunity [48]. Systemic pathogen exposure to *Listeria monocytogenes* and *Salmonella typhimurium* or enteric exposure to *Candida albicans* in inulin-supplemented mice resulted in a reduced mortality compared with cellulose-supplemented controls [51]. A mixture of oligofructose (OF) and high-polymer inulin given orally to rats stimulated SIgA production in the cecum [52]. The oral application of imulin and oligofructose induced the secretion of immunoregulatory IL-10 by ex vivo–activated lymphocytes of Peyer’s patches [53]. γ-inulin enhances humoral and cellular responses against a wide variety of antigens, stimulates Th1 and Th2 lymphocytes, and is a very potent complement activator [54]. In this study, the control group chickens achieved the highest mean body weight at the 35th day of life (2978g) (data not presented) and this differed significantly (*p* < 0.05) in comparison to the Pre1, Pre2, and Syn2 groups (mean body weights of 2447–2515g), but did not differ in comparison to group Syn1 (2926g). This might have been influenced by the highest mortality rate in the control group (Table 3), which caused the removal of the largest number of ill/slowly-growing chickens, than in other groups.

Besides the synbiotics presented in this study, other bioactive substances have also proved their immunomodulatory effects [23,24]. A significant upregulation of gene expression for IL-4, IL-6, IFNβ, and IL-18 and downregulation of IL-12 gene expression were observed in spleens of chickens treated with *L. lactis* subsp. *cremoris* IBB 477 SC1 with raffinose family oligosaccharides (RFO) compared to the control [23]. The same synbiotic (*L. lactis* subsp. *cremoris* IBB SC1 with RFO) injected *in ovo* during embryo development influenced the structure and development of immune organs [24]. The relative spleen weight was significantly higher in chickens treated with *L. lactis* subsp. *cremoris* IBB SC1 combined with RFO and the histological view of the thymus displayed an increase in thymocyte numbers in the cortex.

We did not observe an additive effect of the synbiotic. The same pattern was also observed in the microarray data generated for the cecal tonsils, spleen, and large intestine. The explanation for this phenomenon is related to different routes employed by prebiotics and synbiotics to access the embryo. A soluble prebiotic, injected on the 12th day of embryo development, thanks to the vascularized chorioallantoic membrane, is gradually transferred into the growing embryo. However, probiotic bacteria, due to their size, are unable to penetrate the chorioallantoic membrane. Therefore, they are only consumed by the chicken at the beginning of hatching [28]. An experimental simulation of this hypothesis is presented in the review publication [16]. Prebiotics administered on the 12th day of embryo development stimulate endogenous microbiota of the chicken gastrointestinal tract. Thanks to next generation sequencing, it has been proved that the internal environment of a chicken egg is not sterile [16]. A subsequent experiment on the synbiotic design in vitro and its further validation in vivo showed that the differences in the synbiotic impact on the chicken organism are caused by a mutual interaction between prebiotics and probiotics [18].

Available data have described only selected indicators of immunity, which made the interpretation of our results difficult. Furthermore, the effect of prebiotics or synbiotics in animals depends on many factors, such as the sources of microbiota, doses, frequency of applications, chemical contamination, environmental conditions (elimination of stressors), and the mode and method of administration [12,49,50].

## 5. Conclusions

In summary, the *in ovo* application of prebiotics (inulin or Bi^2^tos) and synbiotics (inulin + *Lactococcus lactis* subsp. *lactis* IBB SL1 or Bi^2^tos + *Lactococcus lactis* subsp. *cremoris* IBB SC1) did not significantly influence the humoral immune response against T-dependent and T-independent model antigens. The *in ovo* application of inulin or Bi^2^tos (in Pre1 and Pre2 groups) in chickens immunized with T-dependent antigen (SRBC) protected them from retarded growth of the IgG concentration, which was observed in the control group on D35. The concentrations of IgG in the yolk sac (from E18 to D21) and blood serum (from D1 to D21) did not differ between groups. In contrast, a significant effect on the DTH reaction to phytohemagglutinin was detected on the 7th (Pre1 < Control) and 21st (Pre2 > Syn2) day after hatching, while no effect was found on day 35th of life. The inulin-treated group (Pre1) exhibited a lower mortality rate (−6.4%) than the control group. It may be concluded that the application of inulin in an egg air chamber on the 12th day of incubation may stimulate the secondary immune response.

## Figures and Tables

**Table 1:** Number of embryos and chickens used in the experiment.

Time	Groups and Number of Embryos/Chickens					Treatment
	Pre1	Pre2	Syn1	Syn2	C	
	900					incubated eggs
E12	−7					randomly selected embryos sacrificed and used before treatment for IgG (Y) estimation
E12	160	160	160	160	160	embryos given to experimental groups and treated *in ovo*
E18	−7	−7	−7	−7	−7	randomly selected embryos from each group sacrificed and used for IgG (Y) estimation
D1	145	137	114	145	130	hatched chickens
D1	−7	−7	−7	−7	−7	randomly selected chickens sacrificed and used for IgG (Y) and IgG estimation
D4	−7	−7	−7	−7	−7	randomly selected chickens sacrificed and used for IgG estimation
D7	−7	−7	−7	−7	−7	randomly selected chickens sacrificed and used for IgG, SRBC, DEX (IgM), and DEX (IgG) antibody estimation
	Groups destined for an evaluation of the serum IgG concentration and DTH test.					
	Pre1(N)	Pre2(N)	Syn1(N)	Syn2(N)	C(N)	
D7	45	42	37	43	32	chickens randomly selected for N groups
D21	−7	−7	−7	−7	−7	randomly selected chickens sacrificed and used for IgG estimation
	−8	−8	−8	−8	−8	randomly selected chickens used for DTH
D35	−7	−7	−7	−7	−7	randomly selected chickens sacrificed and used for IgG estimation
	−8	−8	−8	−8	−8	randomly selected chickens used for DTH

C – control, Pre1 – prebiotic 1, Pre2 – prebiotic 2, Syn1 – synbiotic 1, Syn2 – synbiotic 2, N – nonimmunized groups, IgG (Y) – yolk sac IgG concentration, IgG – serum IgG concentration, SRBC (RAT) – serum reverse agglutination titer against SRBC, DEX (IgM) – serum anti-dextran IgM antibody, DEX (IgG) – serum anti-dextran IgG antibody, and DTH – delayed-type hypersensitivity.

**Table 2:** Chickens immunized against T-dependent (SRBC) and T-independent (dextran) antigens.

Time	Groups and Number of Embryos/Chickens										Treatment
	C SRBC	C DEX	Pre1 SRBC	Pre1 DEX	Pre2 SRBC	Pre2 DEX	Syn1 SRBC	Syn1 DEX	Syn2 SRBC	Syn2 DEX	
D7	25	25	35	35	30	30	25	25	35	35	1^st^ immunization
D21	−7	−7	−7	−7	−7	−7	−7	−7	−7	−7	randomly selected chickens sacrificed and used for IgG, SRBC (RAT), DEX (IgM), and DEX (IgG)
D21	18	18	28	28	23	23	18	18	28	28	2nd immunization
D35	−7	−7	−7	−7	−7	−7	−7	−7	−7	−7	randomly selected chickens sacrificed and used for IgG, SRBC (RAT), DEX (IgM), and DEX (IgG)

C – control, Pre1 – prebiotic 1, Pre2 – prebiotic 2, Syn1 – synbiotic 1, Syn2 – synbiotic 2, SRBC – SRBC-treated groups, DEX – dextran-treated groups, IgG – serum IgG concentration, SRBC (RAT) – serum reverse agglutination titer against SRBC, DEX (IgM) – serum anti-dextran IgM antibody level, and DEX (IgG) – serum anti-dextran IgG antibody level.

**Table 3:** Mortality rate in pre- and symbiotic-treated chickens. C – control, Pre1 – prebiotic 1, Pre2 – prebiotic 2, Syn1 – synbiotic 1, and Syn2 – synbiotic 2.

Groups	Number of Chickens at the Start of the Experiment	Mortality Cases	Mortality Rate (%)
C	130	11	8.5 ^a^
Pre1	145	3	2.1 ^b^
Pre2	137	8	5.8 ^ab^
Syn1	114	6	5.3 ^ab^

^a,b^*p* < 0.05.

## Abbreviations

C/Pre1/Pre2/Syn1/Syn2, control and experimental groups treated at day 12 of embryo development with physiological saline or respective pre- and synbiotics. C DEX/Pre1 DEX/Pre2 DEX/Syn1 DEX/Syn2 DEX, control and experimental groups treated with pre- and synbiotics at day 12 of embryonic development, and later immunized on the 7th and 21st day of life with dextran. C SRBC/Pre1 SRBC/Pre2 SRBC/Syn1 SRBC/Syn2 SRBC, control and experimental groups treated with pre- and synbiotics at day 12 of embryonic development, and later immunized on the 7th and 21st day of life with SRBC. C(N)/Pre1(N)/Pre2(N)/Syn1(N)/Syn2(N), control and experimental groups treated with pre-and synbiotics at day 12 of embryonic development, selected for the DTH skin test. D1 (4, 7, 14, 21, and 35), day of age. DEX, dextran. DTH, delayed-type hypersensitivity. E12 (18), day 12 (18) of embryo development. PHA, phytohemagglutinin. SRBC, sheep red blood cells.
